# Supplementary material for: Brain Death and Its Prediction in Out-of-Hospital Cardiac Arrest Patients Treated with Targeted Temperature Management
Source: Diagnostics (Basel). 2022 May 10;12(5):1190. doi: 10.3390/diagnostics12051190 (PMC9140750; doi:10.3390/diagnostics12051190)
Supplement: Supplementary file 1 [file diagnostics-12-01190-s001.zip › diagnostics-1707855-supplementary.pdf]

Supplementary materials

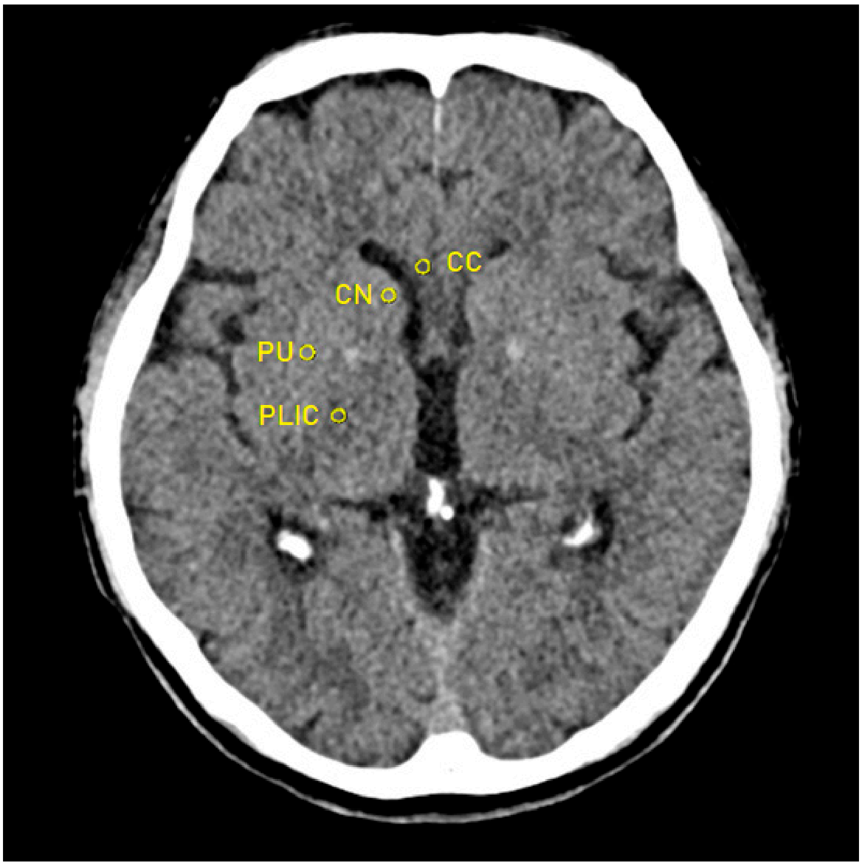

Figure S1. Brain computed tomography images showing the measurements in Hounsfield units at the basal ganglia level. The circular regions of interest (10 mm^2) are positioned in the caudate nucleus (CN), putamen (PU), posterior limb of the internal capsule (PLIC), and genu of the corpus callosum (CC) at the level of the basal ganglia.

The gray-to-white matter ratios (GWRs) were calculated according to the following formulas:

GWR = (CN + PU)/(CC + PLIC)

Table S1. Baseline characteristics and results of prognostication tests in brain-dead patients.

Case	Sex/age, years	Witnessed	Bystander CPR	Shockable rhythm	Anoxic time[a], min	Cause of arrest	GWR	DWI[b]	SSEP	Peak NSE, ng/mL (time, h)	Peak S100, ng/mL (time, h)	Hospital days of BD	Donated organ procurement
1	M/51	Yes	Yes	No	24	Cardiac	1.21	-	N20(-)	98.19 (72)	12.18 (72)	5	Liver, kidneys (2)
2	F/38	No	No	No	41	Asphyxia	1.17	-	N20(-)	201.32 (24)	15.01 (24)	5	Liver
3	F/29	No	Yes	No	110	Asphyxia	1.17	M	N20(-)	72.39 (48)	7.25 (24)	5	Liver, kidneys (2), heart, lung, pancreas
4	M/58	No	No	No	42	Other-medical	1.04	M	N20(-)	201.55 (72)	31.91 (72)	9	Liver
5	M/70	Yes	Yes	No	33	Cardiac	0.90	-	-	207.86 (48)	23.4 (24)	6	Liver, kidneys (2)
6	M/58	Yes	Yes	No	20	Other-medical	1.16	M	N20(-)	209.98 (48)	0.91 (48)	11	Liver, kidneys (2)
7	F/50	No	Yes	No	30	Asphyxia	-	M	N20(-)	91.16 (24)	24.65 (24)	6	Liver, kidneys (2)
8	M/43	No	Yes	No	87	Asphyxia	1.15	-	-	210.97 (24)	5.68 (0)	4	Liver, kidneys (2)
9	M/38	No	Yes	No	53	Asphyxia	1.20	M	N20(-)	136.47(48)	3.87 (0)	4	Liver, kidneys (2), lung, pancreas
10	F/46	No	Yes	No	41	Asphyxia	1.12	M	N20(-)	157.00 (48)	5.38 (0)	6	Liver, kidneys (2), lung
11	M/24	Yes	No	No	52	Asphyxia	1.16	M	-	213.66 (72)	16.8 (72)	7	Liver, kidneys (2), heart, pancreas
12	M/47	No	Yes	No	77	Asphyxia	1.13	M	N20(-)	140.11 (48)	4.67 (0)	11	Liver, kidneys (2), heart, lung
13	M/21	No	Yes	Yes	41	Asphyxia	1.04	M	-	184.13 (24)	12.61 (24)	5	Liver, kidneys (2), heart, pancreas
14	M/45	No	Yes	No	36	Asphyxia	1.18	M	N20(-)	78.56 (24)	30.46 (24)	5	Liver, kidneys (2), heart, pancreas
15	F/33	No	No	Yes	46	Asphyxia	1.18	M	N20(-)	196.67 (48)	0.41 (24)	12	Liver, kidneys (2), lung, heart, pancreas
16	F/38	No	No	No	32	Asphyxia	1.22	M	N20(-)	192.28 (24)	1.51 (24)	7	Liver, kidney, heart, lung, pancreas
17	M/32	No	Yes	No	56	Asphyxia	1.15	M	N20(-)	79.83 (72)	1.25 (0)	7	Liver, kidneys (2), heart
18	M/54	Yes	No	No	54	Cardiac	1.00	M	-	215.0 (24)	15.43 (72)	6	Kidneys (2)
19	M/57	Yes	No	No	41	Other-medical	1.06	M	-	170.58 (24)	18.62 (48)	7	Liver, kidneys (2)

CPR, cardiopulmonary resuscitation; GWR, gray-to-white matter ratio at basal ganglia; DWI, diffusion-weighted imaging; SSEP, somatosensory evoked potential measurements; NSE, neuron-specific enolase; S100B, S100 calcium-binding protein B; BD, brain death.

[a]Time from arrest to return of spontaneous circulation.

[b]DWI findings were categorized as follows: N, no lesion; I, isolated cortex or deep gray matter lesion; M, multifocal or global lesion.
